# The Influence of Short-Range Correlation on the Phonon Confinement of a Single ZnO Nanowire

**DOI:** 10.1186/s11671-017-2013-0

**Published:** 2017-04-08

**Authors:** Po-Hsun Shih, Sheng Yun Wu

**Affiliations:** Department of Physics, National Dong Hwa University, No. 1, Sec. 2, Da-Hsueh Rd., Shoufeng, Hualien, 97401 Taiwan

**Keywords:** Nanocrystalline materials, Confocal Raman spectroscopy, Nanowire

## Abstract

Plenty of researches have been performed to probe the diverse properties of ZnO nanowires, but only a few have focused on the physical properties of a single nanowire since to analyze the optical confinement and their correlation lengths along a single nanowire is difficult. In this study, a single ZnO nanowire was synthesized using a Ti-assisted chemical vapor deposition (CVD) method to avoid the appearance of catalytic contamination. Confocal Raman spectroscopy is a powerful tool for probing the phonon confinement effect in a single ZnO nanowire. A confinement model was used to calculate the correlation lengths along the growth direction. The Raman mapping of ZnO nanowires was obtained by a confocal Raman spectrometer. A phonon confinement model was used to fit the Raman curves of the E_2_ mode and to obtain the correlation lengths along the growth direction of the ZnO nanowire. The correlation lengths are related to the phonon confined region by boundaries and/or defects.

## Background

Zinc oxide (ZnO) nanowires have attracted extensive attentions due to its novel properties as compared with that of bulk. To date, the crystalline ZnO nanowires have been synthesized by various methods such as chemical vapor deposition method using a mixed of ZnO powder and graphite [1], laser ablation method [2], Au-catalyzed vapor transport method [3, 4], and template-directed method using electrochemical deposition technique [5]. Of most various synthesis methods, various catalysts or auxiliaries were used in the fabrication processes. The presence of a residual layer found at the edge or on the surface of nanomaterials increases the difficulty to observe the essential characteristics of ZnO. Therefore, synthesis of nanowires without a catalyst or using one-step growth method [6, 7] would be of interest. These syntheses could be useful to investigate the shifting and broadening of the phonon mode of confinement effects for various sizes of ZnO nanomaterials. Besides the problem of contaminations, another challenge is that the studies of size effects for nanomaterials were usually investigated by examining the physical properties of various size particles. However, the possible contributions from the shape of the nanoparticles, the uniformity of the size distribution, the strain of bonds, and other factors (such as growth conditions and growth techniques) lead to a complicated situation in which the experimental information of the various particle sizes could not be compared with one another [8]. Now, the investigation of a tip-like single ZnO nanowire creates a reasonable possibility for probing the phonon confinement. The gradient of diameters along the length of a tip-like nanowire replaces the effect of various size particles. An optical spatial mapping of confocal Raman spectra permits a direct observation of the nature of an individual nanowire along the growth direction. To the best of our knowledge, plenty of studies have been done to explore diverse properties of ZnO nanowires but only a few have focused on the optical property of a single nanowire. In this study, we reported on the phonon confined effect in a single Ti-assisted ZnO nanowire. The Raman mapping spectra of E_2_ mode of a tip-like nanowire were analyzed. The non-polar E_2_ mode with good signal-to-noise ratio is an appropriate candidate to study the interplay between the local phonon behavior and geometric anisotropy. Owing to the varying diameters being much smaller than the length, the phonon confinement effect of the E_2_ mode of lattice vibration appears in the radial direction [9]. A phonon confinement model was utilized to explain the shifting and broadening of E_2_ mode and to obtain the correlation lengths along the growth direction.

## Methods

The crystalline ZnO nanowires were synthesized using a Ti-grid assisted by a chemical vapor deposition method. No other catalyst and auxiliary were used in this process [10]. The 99.99% zinc grain (0.2 g) on a pure 200 mesh titanium-grid was mounted on a ceramic boat inside a vacuum quartz tube, which was exhausted to about 10^−3^ Torr by a mechanical pump. A mixed gas of oxygen (25%) and argon (75%) was introduced, and the pressure in the tube was kept at near 760 Torr by a gas flow controller. The sample was maintained at 600 °C for 2 h and then cooled down to room temperature naturally, after which the sample was reserved in a vacuum chamber to avoid further oxidation. In this Ti-grid-assisted CVD method, Ti, Zn, and ZnO have hexagonal structures and similar lattice constants. Based on this reason, the Ti grid was used as a substrate. The diameter and length of ZnO nanowires can be adjusted by controlling the annealing temperature, growing time, and the level of working pressure. Field-emission scanning electron microscopy (FESEM; Jeol JSM-6500F) equipped with a wavelength dispersive spectroscopy (WDS) and with an energy dispersive spectroscopy was employed to characterize the morphology of as-grown ZnO nanowires.

## Results and Discussion

### Sample Characterization

**Fig. 1:**
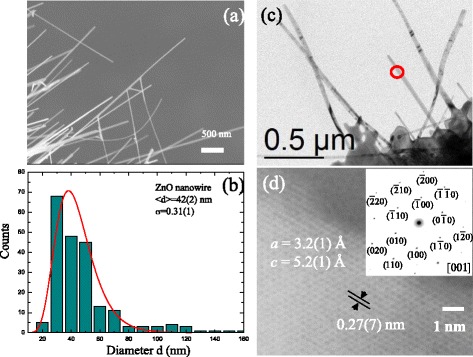
**a**, **b** SEM image and diameter distribution for ZnO nanowires synthesized at 600 °C. **c**, **d** TEM image and high-resolution image of the selection region of ZnO nanowires. *Inset* displays a corresponding selected-area electron pattern

Figure 1a displays the SEM image showing the morphology of ZnO nanowires. It can be seen that the ZnO nanowires grew homogeneously in a large area of the Ti grid substrate to form straight nanowires. The diameters ranged from 20 to 50 nm, and the lengths were up to several 10 μm. The distribution of nanowires obtained from a portion of the SEM image is quite asymmetric and can be described using a log-normal distribution function. We measured the diameters for various samples and fitted the curves using a log-normal function respectively. The log-normal function is defined as follows: $f(d)=\frac{1}{{\left(2\pi \right)}^{0.5} d\sigma} \exp \left[-\frac{{\left( \ln d- \ln < d>\right)}^2}{2{\sigma}^2}\right]$, where *d* is the diameter, <*d*> is the mean value, and σ is the standard deviation. The mean diameter obtained from the fits is <d> = 42(2) nm, and the standard deviation is σ = 0.31(1); the width of the distribution profile is presumably broadened due to the crystalline and oxidation temperature effects. Transmission electron microscopic (TEM) images and high-resolution transmission electron microscopic (HRTEM) images taken from a JEM-3010 analytical transmission electron microscopy (Jeol, Japan) were obtained to study the crystalline structure, as shown in Fig. 1c, d, respectively. The TEM result reveals an individual straight nanowire, and the diameter along the growth direction is relatively uniform, with a mean value of 45(5) nm, which is close to the value of SEM results. The corresponding selected-area electron diffraction (SAED) pattern shown in the inset of Fig. 1d, taken from a single nanowire, reveals the single crystalline nature of the sample. The Bragg diffraction spots correspond to the [001] reflection of the hexagonal structure of ZnO nanowires, and the pattern of main spots can easily be indexed based on a hexagonal structure with lattice parameters of *a* = *b* = 3.2(1) Å and *c* = 5.2(1) Å.

### Conceptual Design of Confocal Raman Mapping

**Fig. 2:**
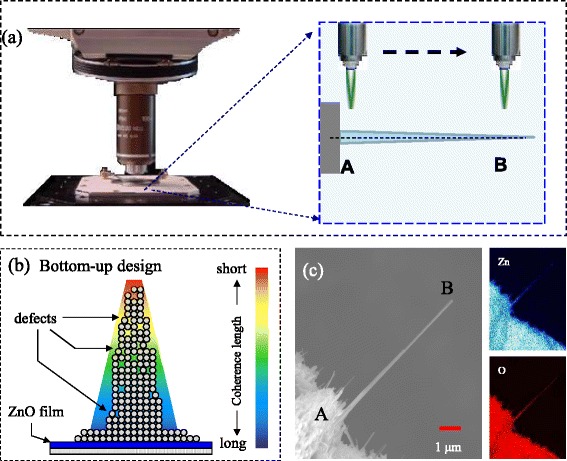
**a** Schematic diagram of the Raman spatial mapping that is scanned from A (root) toward B (tip) along a single ZnO nanowire. **b** Conceptual diagram of defect distribution in ZnO nanowire. **c** SEM image of the selected ZnO nanowire at magnification of 10,000 and (*insets*) the corresponding WDS mappings of zinc and oxygen elements from A (root) to B (tip), respectively

Confocal Raman spectrometer (Wietec Alpha 300) was used to investigate the spectroscopic properties of an individual ZnO nanowire, recording backscattering geometries with no polarization direction. Confocal Raman scattering with high spatial resolution corresponding to half the wavelength of the exciting light is an advanced technique for examining the phonon confinement effect along a single nanowire. From an experimental point of view, ZnO crystallization occurs in a wurtzite hexagonal structure with a space group of *P6*_*3*_ *mc*. Theoretically, there is a total of 12 phonon modes, in which the low- and high-frequency E_2_ mode are respectively associated with the vibration of the heavy zinc atoms and light oxygen atoms. The theoretical calculation for high-frequency E_2_ mode is 433 cm^−1^ [11]. In a nanocrystalline system, the size dependency of the energy shift and the broadening of the profile could be caused by particle shapes, size distributions, bond strains, thermal effects, and surface enhancements. The multiple factors result in an obscure situation for investigating the size effect. In order to obtain a better interpretation of experimental data and avoid the uncertainties, confocal Raman spectroscopy was performed on a tip-like ZnO nanowire. The conceptual design was plotted schematically in Fig. 2a. It can be seen that, for the tip-like single nanowire grown on the Ti-grid surface, the diameters of a cylindrical cross section decrease with the increasing length to form a gradient of diameters along the length of the nanowire. A typical ZnO nanowire has a length up to several micrometers that are greater than the varying diameters along the nanowire. It means that the phonon confinement effect mainly occurs in the radial direction. Different colors drawn on the nanowire are used to differentiate the levels of correlation lengths related with the distances between defects. Figure 2b shows a schematic illustration of the defect distribution in a single ZnO nanowire. As far away from the substrate, the crystalline quality becomes poor and the defect density increases, revealing that the correlation length decreases with decreasing diameter. In order to confirm this assumption, a typical tip-like ZnO nanowire was selected as shown in Fig. 2c. The selected ZnO nanowire in this investigation was ~7.29 μm long. The diameters at the top and the bottom were respectively 10 and 250 nm, revealing that was a cylindrical nanowire. The insets depict the fixed X-ray energy of zinc (L_α_) and oxygen (K_α_) analyzed by WDS mapping, respectively. The blue and red dots reflect the existence of zinc and oxygen atoms, respectively. Obviously, the results reveal a gradient of distribution on the tip-like nanowire with high density of Zn and O at the bottom and with low density at the top, while the Ti signals (data not shown) were hard to be detected due to the zinc oxide film. The WDS result confirms that the tip-like nanowire was composed of zinc and oxygen elements and the titanium was only an auxiliary. This can help us to investigate the phonon confinement effect in a single nanowire without considering the effect of any residual material.

### Confocal Raman Scattering

**Fig. 3:**
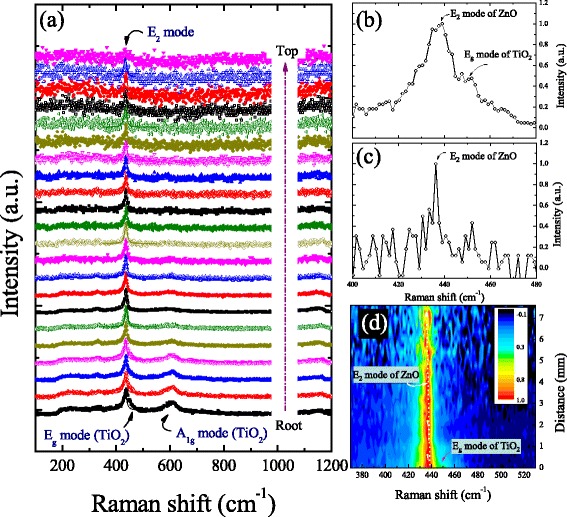
**a** A series of Raman spectra of E_2_ mode taken along the growth direction. Corresponding Raman spectra **b** at the bottom and **c** at the top of the tip-like ZnO nanowire. **d** Two-dimensional map of intensity and length dependence of Raman spectra taken at room temperature

Prior to the Raman mapping, the sample was mounted on a highly linear piezo-driven feedback controlled scanning stage. The single ZnO nanowire was excited with a 488-nm Ar ion laser with 5 mW laser power on the sample, to form a spot size of ~0.3 μm in diameter. No significant difference of Raman shifts was found during the laser annealing. The Raman spectra of the tip-like single ZnO nanowire were scanned from the base at the point A along the growth direction to point B as shown in Fig. 2c. Figure 3a shows a series of Raman spectra made at various points along the scanning direction. The Raman spectra reveal two main phonon modes in the bottom of the tip-like ZnO nanowire, as shown in the bottom of Fig. 3b, at 437.5 and 447 cm^−1^, corresponding to the E_2_ (high frequency) symmetry of ZnO and to E_g_ symmetries of TiO_2_ [12], respectively. For the first four spectra, the asymmetric broad peak at 438 cm^−1^ should be attributed with a boundary transition between ZnO_1−x_ and ZnO phases and with a near peak of E_g_ mode of TiO_2_ originated from the substrate. A similar observation of boundary transition by EDS mapping for CuO nanowires has been reported by Cheng et al. [13]. Along the growth direction, as far away the substrate, the intensity of A_1g_ mode of TiO_2_ decreased clearly. After ~1 μm, for the main peak, the contribution of E_g_ mode (TiO_2_) from the substrate was assumed to vanish based on the disappeared peak of A_1g_ mode of TiO_2_. It means that the Raman spectra taken from 1 to 7 μm reflect the Raman curves without being affected by substrate interference. At the top of nanowire as shown in Fig. 3c, only one unclear peak centered at 436.3 cm^−1^ corresponding to E_2_ mode of ZnO could be observed. The poor signal-to-noise ratio of Raman spectrum taken from the top of tip-like nanowire is due to that the nanowire may not be parallel to the scanning direction of the detector. It can be predicted that scanning along a tilled nanowire would be out of focus, especially for confocal Raman scattering. Another possible reason for the weak intensity is that the tip volume of a tip-like nanowire is smaller than the root volume. It is known that the intensity of Raman scattering is proportional to the number of scattering centers present in the volume illuminated by the laser, so the intensity is decreasing with decreasing diameters along the nanowire. According to Fig. 3d, overall, the summed intensity at lower frequency of the asymmetric peak centered at around 436 cm^−1^ is larger than that at the higher frequency, revealing the existence of phonon confinement effect that results in the asymmetric broadenings. Furthermore, there is seemingly a non-clear dependence of E_2_ mode frequency with diameters while the peak-widths vary with various positions along the nanowire, which is similar with previous reports for ZnO nanoparticles [14, 15]. Comparing with previous work of ZnO nanowires [16], Huang et al. reported that two peaks of E_2_ mode taken respectively from tips and roots of ZnO nanowires/belts arrays were almost centered at the same position of 436 cm^−1^. These previous studies reveal a phenomenon that, for E_2_ mode of ZnO, the existence of phonon confinement effect for various sizes could be observed on the dependence of asymmetric broadening but not be easily found on that of the frequency shift.

### Phonon Confinement Effect

**Fig. 4:**
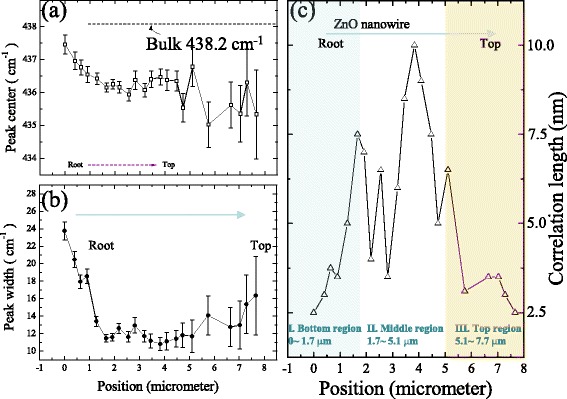
**a** Peak positions, **b** widths, and **c** correlation lengths versus the nanowire length along the tip-like ZnO nanowire

**Fig. 5:**
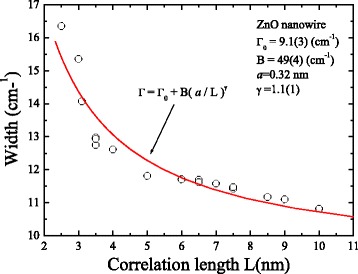
Plot of calculated values of correlation length versus profile width. The *red* solid curve indicates the fit of the data to the theoretical curve for a decay function

To further characterize and interpret the difference in the Raman spectra along a tip-like single ZnO nanowire, a well-known phonon confinement model proposed by Richter et al. [17] was utilized. This model depicts the crystalline quality or boundary size by introducing a parameter known as correlation length. In an ideal lattice, due to the momentum conservation, Raman intensity is related to contributions from phonons with a wave vector (q~0) near the center of the Brillouin zone. But, in a finite region, phonons confined by crystal boundaries or structural defects [18–21], because of the relaxation of the selection rule (q ≠ 0), lead to an additional contribution at the low-frequency side, resulting in an asymmetric broadening of Raman scattering. In order to verify the confinement effect in the tip-like ZnO nanowire, the evolution of the E_2_ mode is taken in the following analysis to calculate the correlation lengths. According to the model, the first-order Raman intensity can be written as: $I(w)\cong A{\displaystyle \underset{BZ}{\int}\frac{{\left| C\left(0, q\right)\right|}^2}{{\left( w- w(q)\right)}^2+{\left(\frac{\varGamma_0}{2}\right)}^2}{d}^3 q}$, where *A* presents an amplitude factor, *q* is the wave vector, *Γ*_0_ is the natural line width, *w*(*q*) is the dispersion relation, and |*C*(*o*, *q*)|^2^ is the Fourier coefficient of the phonon confinement function. Taking the Gaussian confinement function, the Fourier coefficient |*C*(*o*, *q*)|^2^ for a column shaped crystal can be evaluated for given correlation length L and axis length [22] as ${\left| C\left(0, q\right)\right|}^2= \exp \left(-\frac{q^2{L}^2}{\hbox{'}4{\pi}^2}\right)$. As the correlation length decreases, the Raman peak is expected to shift toward lower frequencies and the line shape will become asymmetric with a low-frequency tail. In general, the correlation length can be treated as the average size of the localized region [23]. An analogous form of Fourier coefficient for column-shaped crystals was offered by Wang et al. [9]. In this previous paper, the coefficient was also applied for that the confinement effect occurs along the diameter direction. Incidentally, a modified calculation for nanoparticles proposed by Meier et al. [24] is not useful in this system due to that the size distribution factor could not be considered. On the other hand, according to the ab initio phonon dispersion, the dispersion relation can be expressed as the following equation [20]: *w*(*q*) = *A* + *B* cos(*πq*), which with *A* = 424.5 cm^−1^ and *B* = 12.5 cm^−1^ for the E_2_ (high) mode. Figure 4a, b shows the peak shifts and peak widths versus the nanowire length, respectively. The values of shifts and widths were obtained by fitting the curves using a Lorentz function [25]. As shown in Fig. 4a, a slight red shift could be observed in a limited range of 435–437 cm^−1^. Comparing with the bulk (located at 438.2 cm^−1^), this shift was associated to the size effect [19, 26]. The increasing uncertainty was due to the decreasing single-to-noise ratio. The distance dependence of profile width for E_2_ mode was shown in Fig. 4b. It can be seen that the profile could be clearly divided into three regions, in which two turning points located respectively at 1.7 and 5.1 μm. The rapidly decreasing width below 1.7 μm was attributed to the reduced influence of boundary transition. In the middle of ZnO nanowire, the slight fluctuation in line width implies a relatively stable crystallization. After ~5.1 μm, the line width increased until the end of nanowire. The broadening width could be introduced as the reduced correlation length is due to the structural defects [18]. Figure 4c displays the correlation lengths by fitting the Raman curves using the phonon confinement model. The series of spectra have been fitted in a wavenumber range of 350–550 cm^−1^. The fitting curves were plotted as solid lines in Fig. 3a. In the first bottom region (from 0 to 1.7 μm), the phonon was presumably affected by the transition phase (ZnO_1−x_/ZnO) and was confined in a very small spatial volume. As far away from the boundary of mixed phase, along the growth direction, the distances between defects decreased rapidly and the correlation lengths increased certainly from 2.5 to 7.5 nm. In the second middle region (from 1.7 to 5.1 μm), the correlation lengths were in a limited range of 3.5–10 nm. A maximal value could be observed at the length of 3.83 μm. After that, the correlation lengths decreased as the diameters reduced along the nanowire length. The clear fluctuation could be observed, but, compared with other regions, this region was relatively ordered and defectless. In the third top region (from 5.1 to 7.7 μm), the correlation lengths decreased by ~62% (from 6.5 to 2.5 nm) within less than 3 μm along the nanowire length. The obvious trend reveals an increased defect density and a more disordered crystal in the top of the single ZnO nanowire. The correlation lengths are clearly smaller than diameters, so a usual explanation that phonon confined by boundaries is not suitable in this system. Besides, a common formula was used to describe the relation between the correlation lengths and the broadening profile widths as the following equation: *Γ* = *Γ*_0_ + *B*(*a*/*L*)^*r*^, where *Γ*_0_ is the width for bulk, *a* is the lattice constant, *L* is the obtained correlation length, and *B* and *γ* are adjustable parameters. The *γ* value is assumed to be near to 1.5 and 1 for spherical and column nanomaterials in an infinite size, respectively [27]. The fitted curve as shown in Fig. 5 is quite consistent with the data. The fitting result of *γ* (=1.1(1)) is close to experimental result of 1.0 for silicon columns, respectively. The discrepancy might be originated from non-uniform defect density [12]. Overall, these results were different with that for investigation of another optoelectronic material of TiO_2_ [12], in which clear shifts of frequency and bandwidth of E_g_ mode for various size nanoparticles were found in Raman analysis. In related researches for ZnO, our data is different from the report [28] that E_2_ mode displays no obvious size dependence within a diameter range of 5.7–500 nm. However, in this report, the clear broadening widths and the related correlation lengths have not been discussed. Actually, rare reports have deeply discussed that the correlation lengths were inconsistent with the nanoparticle sizes. In the investigation of GaF_2_ nanoparticles [25], Ricci et al. found a discrepancy between correlation lengths obtained by phonon confinement model and diameters found by XRD analysis. They inferred that the surface layer with high defect density surrounded the crystallite nucleus, so the correlation lengths were shorter than the diameters. In our case, we suggested that the defect density increased with the diameter decreased along the tip-like ZnO nanowire. In other words, the phonons were confined in a gradually smaller volume which led to decrease correlation lengths and broaden the peak widths.

**Fig. 6:**
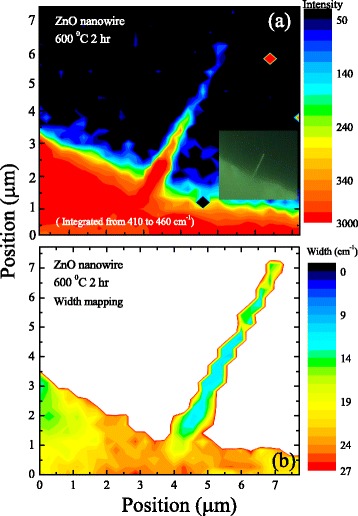
**a** Raman intensities mapping of ZnO E_2_ peak ranging from 410 to 460 cm^−1^ and (*inset*) the corresponding optical image. **b** The corresponding selected area width mapping with a size of ~7.7 μm

The integrated intensities corresponding to the E_2_ mode were mapped and displayed in Fig. 6a for a 7.7 × 7.7 μm^2^ area. The various colors offer a direct visualization of the spatial distribution which is easy to connect to the corresponding optical image shown in the inset. The red area in the lower left corner of the figure revealed the existence of ZnO film on the Ti-grid, in agreement with the previous WDS observation. Along the growth direction of the tip-like ZnO nanowire, the integrated intensities gradually reduced due to the decreased scattering area that we have mentioned in previous paragraph. Moreover, the surface roughness increases as the diameter decreases. The intensity decreased by a factor of 2 when the nanowire length exceeded 3.8 μm. In addition, a few abnormal spots in the right half of the figure were referred to unexpected background noise. Figure 6b displays a corresponding selected area width mapping with size of 7.7 × 7.7 μm^2^. The gradient color is utilized to present the various widths. The colorful substrate may be attributed with the disordered ZnO film. Along the growth direction of the ZnO nanowire, the width reduced with the decreasing diameter as shown in Fig. 4b. It is worth to note that, in the radial direction, the width decreases from the center of ZnO nanowire to the surface. This appearance is assumed to be associated with the nanowire boundary and the disordered surface [9, 29]. Near the surface and edge of ZnO nanowire, the Raman line was affected not only by phonon confinement but also by the non-stoichiometry effect [30]. The peak width is approximately opposite to the correlation length, so the width mapping, as shown in Fig. 6b, offers an indirect but convenient way to depict a rough mapping of correlation lengths in a single nanowire. The discrepancy of correlation lengths in the mapping of ZnO nanowire could be explained by suggesting that the ZnO is consisted of a collection of crystalline grains due to structural defects and the grain size decreases (i.e., the defect density increases) along the growth direction.

## Conclusions

In summary, a tip-like single ZnO nanowire was utilized to investigate the phonon confinement effect. We have fabricated tip-like ZnO nanowires by employing the Ti-assisted CVD method. Confocal Raman scattering with high spatial resolution was performed. The slight frequency shift and the clear line-width broadening of non-polar E_2_ mode of ZnO were attributed with the phonon confinement effect. The correlation lengths related to the average distance between defects were obtained by fitting Raman spectra using a phonon confinement model. The results indicate that, along the growth direction, the correlation lengths reduced with decreasing diameters, revealing that the top region with smaller diameters has a higher defect density.

## Abbreviations

CVD: Chemical vapor deposition
FE-SEM: Field-emission scanning electron microscopy
HRTEM: High-resolution transmission electron microscopy
SAED: Selected-area electron diffraction
TEM: Transmission electron microscopy
WDS: Wavelength dispersive spectroscopy
